# Less demand on stem cell marker-positive cancer cells may characterize metastasis of colon cancer

**DOI:** 10.1371/journal.pone.0277395

**Published:** 2023-04-25

**Authors:** Takeshi Kaida, Yoshiki Fujiyama, Takafumi Soeno, Mitsuo Yokota, Shuji Nakamoto, Takuya Goto, Akiko Watanabe, Kota Okuno, Yusuke Nie, Shiori Fujino, Kazuko Yokota, Hiroki Harada, Yoko Tanaka, Toshimichi Tanaka, Keigo Yokoi, Ken Kojo, Hirohisa Miura, Takahiro Yamanashi, Takeo Sato, Jiichiro Sasaki, Takafumi Sangai, Naoki Hiki, Yusuke Kumamoto, Takeshi Naitoh, Keishi Yamashita

**Affiliations:** 1 Department of Surgery, Kitasato University Graduate School of Medical Sciences, Sagamihara, Kanagawa, Japan; 2 Department of Lower Gastrointestinal Surgery, Kitasato University School of Medicine, Sagamihara, Kanagawa, Japan; 3 Department of General Pediatric and Hepatobiliary Pancreatic Surgery, Kitasato University School of Medicine, Sagamihara, Kanagawa, Japan; 4 Department of Upper Gastrointestinal Surgery, Kitasato University School of Medicine, Sagamihara, Kanagawa, Japan; 5 Department of Breast and Thyroid Surgery, Kitasato University School of Medicine, Sagamihara, Kanagawa, Japan; 6 Multidisciplinary Cancer Care and Treatment Center, Kitasato University School of Medicine, Sagamihara, Kanagawa, Japan; 7 Division of Advanced Surgical Oncology, Research and Development Center for New Frontiers, Kitasato University School of Medicine, Sagamihara, Kanagawa, Japan; CNR, ITALY

## Abstract

**Background:**

CD44 and CD133 are stem cell markers in colorectal cancer (CRC). *CD44* has distinctive isoforms with different oncological properties like total *CD44 (CD44T)* and variant *CD44 (CD44V)*. Clinical significance of such markers remains elusive.

**Methods:**

Sixty colon cancer were examined for *CD44T/CD44V* and *CD133* at mRNA level in a quantitative PCR, and clarified for their association with clinicopathological factors.

**Results:**

(1) Both *CD44T* and *CD44V* showed higher expression in primary colon tumors than in non-cancerous mucosas (p<0.0001), while *CD133* was expressed even in non-cancerous mucosa and rather decreased in the tumors (p = 0.048). (2) *CD44V* expression was significantly associated with *CD44T* expression (R = 0.62, p<0.0001), while they were not correlated to *CD133* at all in the primary tumors. (3) *CD44V/CD44T* expressions were significantly higher in right colon cancer than in left colon cancer (p = 0.035/p = 0.012, respectively), while *CD133* expression were not (p = 0.20). (4) In primary tumors, unexpectedly, *CD44V/CD44T/CD133* mRNA expressions were not correlated with aggressive phenotypes, but *CD44V/CD44T* rather significantly with less aggressive lymph node metastasis/distant metastasis (p = 0.040/p = 0.039, respectively). Moreover, both *CD44V* and *CD133* expressions were significantly decreased in liver metastasis as compared to primary tumors (p = 0.0005 and p = 0.0006, respectively).

**Conclusion:**

Our transcript expression analysis of cancer stem cell markers did not conclude that their expression could represent aggressive phenotypes of primary and metastatic tumors, and rather represented less demand on stem cell marker-positive cancer cells.

## Introduction

Colorectal cancer (CRC) is the recent leading cause of deaths among human cancers [1]. Conventional type of CRC is a genetic disease, and genomic mutations are the most frequent among human cancers [2]; mutations of *APC (Adenomatous polyposis coli)*, *K-ras*, and *TP53 (Tumor protein p53)* are uniquely frequent in 81%, 43%, and 60%, respectively, by new generation sequence (NGS). Molecular target therapy had succeeded according to *K-ras* mutation status in CRC [3]. The latest promising preclinical studies also revealed that APC/β-catenin pathway could be targeted by TNIK (TRAF and NCK-interacting protein kinase) inhibition [4], while gain of function of *TP53* is also an emerging potential target for tumor cells with *TP53* mutation [5, 6].

On the other hand, an alternate target strategy of CRC was based on the cancer stem cell theory [7]. Cancer stem cells are supposed to be resistant to the various anti-cancer therapies and an obstacle against treatments, and stem cell biomarkers have been identified such as *CD44*, *CD133*, *Ascl2*, and *Lgr5* in CRC [8–10].

Ishimoto et al. demonstrated that among the various *CD44* transcripts, only *CD44 variants (CD44V) including CD44v9* can interact with xCT (cystine transporter) through *CD44v9* exon counterpart and established a function for CD44V in regulation of ROS (reactive oxygen species) as an antioxidant molecule. CD44V can bind with xCT to augment cystine influx into cytoplasm, leading to high concentration of intracellular cysteine, and tumor growth through cysteine transportation was demonstrated to play an important role in cancer stemness (cancer-initiation capacity) in the context of its NRF2 (NF-E2-related factor 2) association [11, 12].

Interestingly, *CD44V* expression is frequently seen in primary CRC tissues, while non-cancerous colon mucosa tissues expressed only CD44S (standard form with no variants sequence) [13, 14]. *CD44V* expression was the most early proposed to be a cancer progression marker in CRC [15, 16], whereas it finally failed to demonstrate it [17, 18]. As a result, an interest in *CD44* expression profiles as progression markers of CRC had gradually declined, and has not been sufficiently validated yet at present. Such controversial issues are afterwards repeated even in the quantitative analysis [19, 20].

Allowing for the recent advanced understanding of *CD44* variant isoforms, we hypothesized that clinical significance of *CD44V* putatively representing cancer stemness properties may be different from *CD44T*. In this study, we for the first time developed real time PCR for *CD44v8-v10* as the most optimal representative for *CD44V* expression together with *CD44T* expression and an emerging stem cell marker *CD133* in primary colon tumors, because such differential quantitative assessments have never been done in primary colon cancer. Therefore, we thought that we could report new findings by developing quantitative assessment systems, especially for the *CD44* variant form.

## Materials and methods

### Patients

We collected both tumor and corresponding non-cancerous colon mucosa specimens from 60 consecutive colon cancer patients who underwent colectomy at the Department of Lower Gastrointestinal Surgery, Kitasato University Hospital, Japan, from January 2018 through December 2018. We also collected 33 liver metastasis specimens from 22 consecutive CRC cancer patients (including 8 colon cancer) with liver metastasis who underwent liver resection at the Department of General Pediatric and Hepatobiliary Pancreatic Surgery, Kitasato University Hospital, Japan, from June 2020 through December 2020. Colon cancer patients were classified by the 8^th^ TNM classification of the Union for International Cancer Control (UICC). All tissues were collected at the Kitasato University Hospital, and informed consent was obtained. Informed consent was obtained in writing with the patient’s consent. This study was performed with approval of the Ethics Committee of the Kitasato University School of Medicine (approve number B20-382).

### Cell lines and cultures

We used CRC cell lines; COLO205, COLO320, DLD1, HCT116, and LOVO were provided by the Cell Response Center for Biomedical Research Institute of Development, Aging and Cancer, Tohoku University (Sendai, Japan), SW480 cells were purchased from the American Type Culture Collection (ATCC) (Manassas, VA, USA). HCT15 cells were purchased form the RIKEN BioResouce Center (Tsukuba, Japan), and LS174T cells were purchased from the European collection of authenticated cultures (Porton Down, Salisbury, UK). LOVO and SW480 were grown in mixed medium (1:1 of RPMI1640: F-12 HAMS purchased from Sigma-Aldrich N6658, St. Louis, MO, USA) and Leibovitz L15 medium (ThermoFisher SCIENTIFIC 11415064, Walltham, MA, USA), while all other cell lines were grown in RPMI1640 medium (Gibco, Carlsbad, CA, USA), supplemented with 10% fetal bovine serum and penicillin-streptomycin (Gibco).

### RNA extraction and cDNA generation

During surgery, all specimens were immediately collected and stored in RNAlater^TM^ RNA Stabilization Solution (ThermoFisher SCIENTIFIC, Invitrogen) at 4 degree overnight. The next day, the specimens were sliced down and stored at -80 degree. RNA extraction from homogenized specimens and cell lines was performed using RNeasy Mini Kit (Qiagen) following the manufacture’s protocol. Two micrograms of total RNA were reversed transcribed into complementary DNA (cDNA) using oligo-d(T) primer and the SuperScript Ⅲ reverse transcriptase kit (ThermoFisher SCIENTIFIC, Invitrogen).

### Semi-quantitative RT-PCR

**Fig 1:**
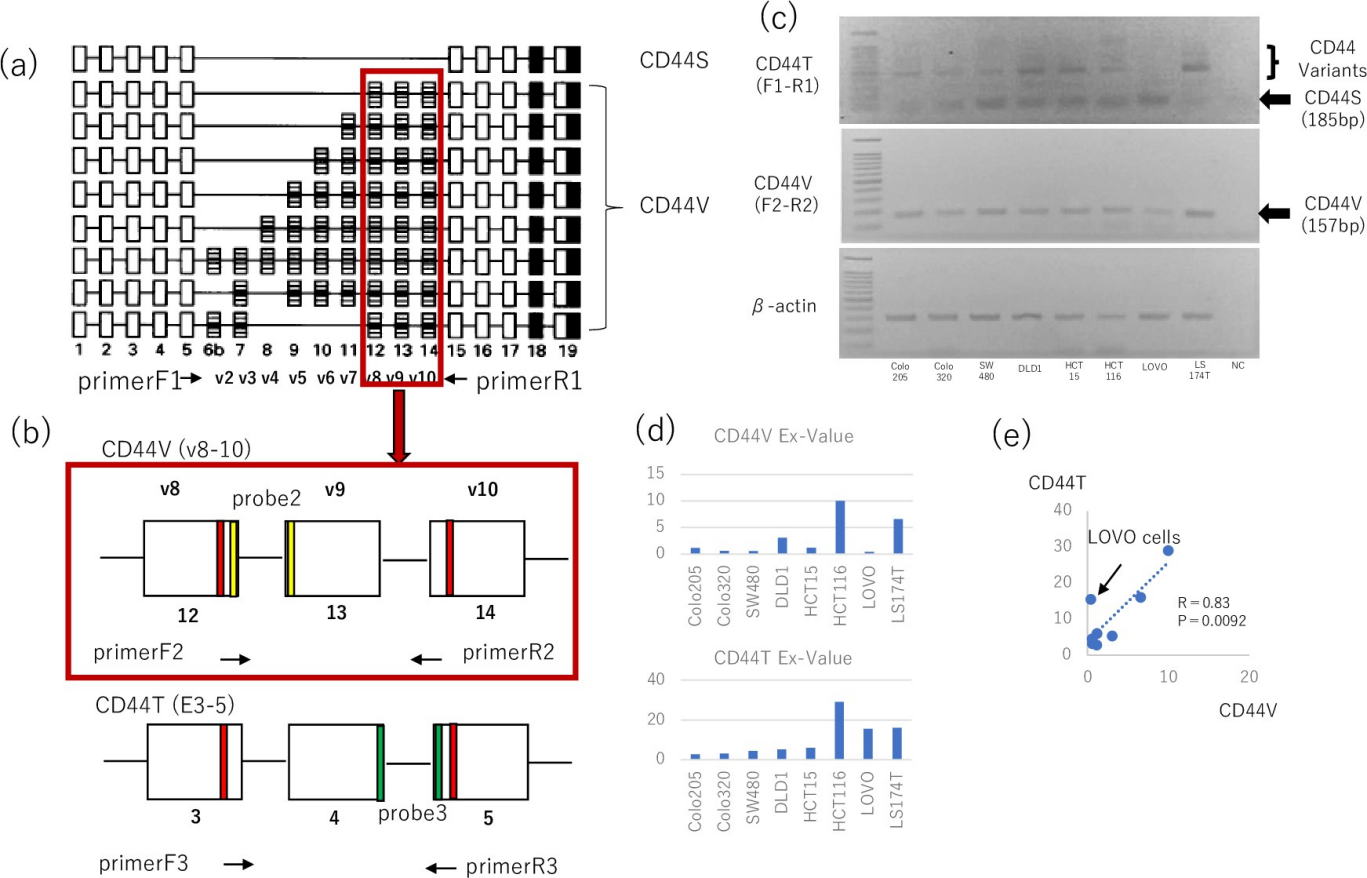
*CD44* transcripts expression in CRC cell lines. (a) *CD44* transcripts in CRC included both CD44S (standard) and CD44V (variants), and CD44V always included variant 8–10 from the reference 17. PrimerF1 and primerR1 can amplify both CD44S and CD44V. (b) For CD44V (v8-10), primerF2 and primerR2 were selected on the variant 8 and variant 10, respectively, with probe 2. On the other hand, for CD44T (total CD44), primerF3 and primerR3 were selected on the exon 3 and exon 5, respectively, with probe 3. (c) In semi-quantitative RT-PCR (F1-R1), multiple bands representing CD44 Variants and CD44S (185 bp) were seen in 8 CRC cell lines (upper panel). Using F2-R2 primer sets, CD44V (157 bp) could be amplified as a single band (middle panel). The lower panel showed β-actin expression as internal controls. (d) In quantitative real time PCR, CD44V and CD44T transcripts were measured in 8 CRC cell lines. Transcriptional discrepancy was seen in LOVO cells. (e) In CRC cell lines, there were close association (R = 0.83, P = 0.0092) of transcripts between CD44T and CD44V.

The pooled assay mix contained primers at a final concentration of 0.2 mL, 1mL of dNTP mixture, 1.5mL of MgCl_2_, 5 mL of PCR buffer, and 0.2 mL of Platinum Taq DNA polymerase (Invitrogen) in a final volume of 50mL. The thermal cycling conditions for *CD44T* and *CD44V* were an initial hold at 95 degree for 3 min and 35 cycles of 30 second at 95 degree, 30 second at annealing temperature (60 degree), 30 second at 72 degree, and 10 min at 72 degree. Both Primer F1 and Primer R1 were designed for gene amplification from exon 5 to exon 15 (Fig 1A), and both Primer F2 and Primer R2 were made for gene amplification from exon 8 to exon 10 (Fig 1B). The primer sequences are shown as follows (S1 Table).

### Quantitative RT-PCR (Q-RT-PCR)

Gene expression levels were assessed by Q-RT-PCR with iQ^TM^ Supermix Real-Time PCR Detection System (Bio-Rad, Hercules, CA, USA). PCR conditions for *CD44V (v8-10)* (Fig 1B) were an initial hold at 95 degree 3 min and 40 cycles of 20 second at 95 degree, 30 second at annealing temperature (60 degree), 30 second at 72 degree in a 25 mL reaction volume containing 200 nmol/L fluorescent probe (probe 2) and 12.5 mL iQ^TM^ Supermix. Target gene expression levels (Ex-Value through the text) were normalized to β-actin expression and analyzed using the comparative cycle threshold method (Ex-value was defined as target gene expression/ β-actin expression x 100).

Variant 9 was encompassed by primers of both variant 8 and variant 10 designed for quantitative PCR for *CD44V*, whereas *CD44T* was represented by intracellular structure for CD44 commonly with all isoforms shared.

### Statistical analysis

Continuous variables were evaluated using the Student’s *t* test, and categorical variables were evaluated using Fisher’s exact test or Chi-square test, as appropriate. A p value below 0.05 was considered to indicate statistical significance. All calculations were performed using JMP version 14 software (SAS institute Japan, Tokyo, Japan).

## Results

### *CD44* expression in CRC cell lines

*CD44* transcripts from CRC tumors are comprised of various isoforms including both *CD44S* and *CD44V* using pooled colonic tumor tissues by sequencing as indicated in Fig 1A as previous report [17], and interestingly all variant forms of *CD44V* inevitably included exon8-10. *CD44V* encompassed v9 exon which includes a region bound with cysteine transporter xCT to regulate redox status in cancer cells and sustain stemness [11]. Thus, *CD44V* is considered to represent cancer stemness.

Using both PrimerF1 and PrimerR1 (Fig 1A), *CD44 Variants* (multiple bands) were detected in CRC cell lines except LOVO cells, while *CD44S* was also expressed in CRC cell lines except LS174T (top panel of Fig 1C). Early reports described that *CD44S* was detected only in non-cancerous mucosas [21], however almost CRC cell lines clearly expressed it in our current study.

Using both PrimerF2 and PrimerR2 (Fig 1B), *CD44V* (v8-v10) which may represent total amounts of variants in a single band were strongly expressed in CRC cell lines except LOVO cells, which deemed consistent with *CD44* Variants made by primerF1/primerR1 (middle panel of Fig 1C). β*-actin* was also shown as an internal control (bottom panel of Fig 1C).

Quantitative real time RT-PCR was then developed for *CD44V* and *CD44T* using their specific primers (primerF2/R2 and primerF3/R3, respectively) with TaqMan probes (probe2 and probe3, respectively) (Fig 1D), and HCT116 cells were proved to show the strongest bands for both forms and used as a positive control for *CD44V/T* expression.

Real time RT-PCR confirmed that the intense expression of *CD44V* was confirmed to be recognized in HCT116, LS174T, and DLD1 cells in order of expression level, and that LOVO cell was also additional for *CD44T* expression. In LOVO cells, *CD44S* is predominantly expressed differently from other cell lines. Interestingly, there was close association in expression between *CD44V* and *CD44T* in CRC cell lines except LOVO cells (R = 0.83, p = 0.0092, Fig 1E).

### *CD44V/CD44T* expression in primary colon cancer

**Fig 2:**
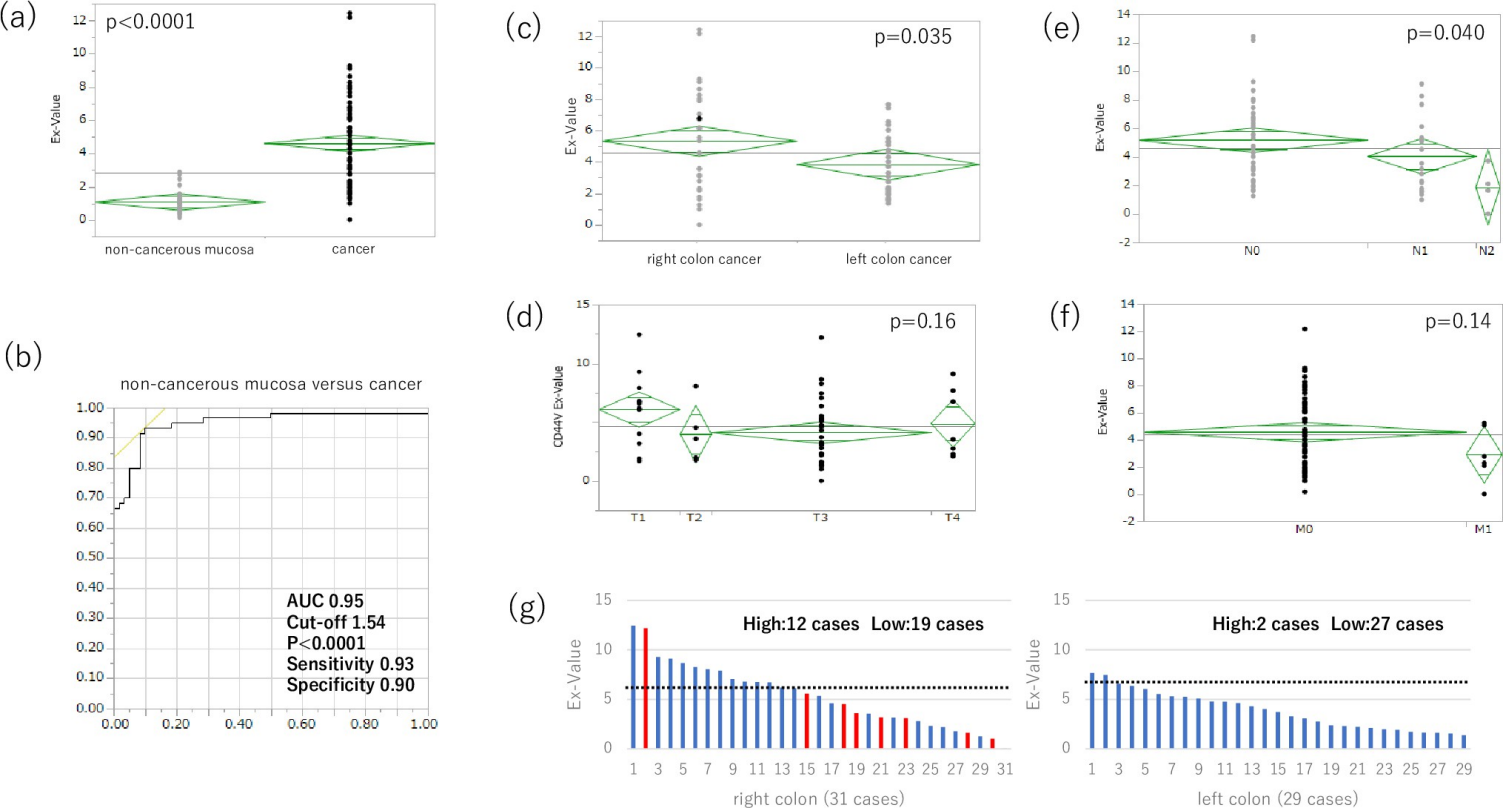
Clinical significance of CD44V expression in primary colon cancer tissues. (a) CD44V expression is elevated in 60 primary colon cancer tissues as compared to in 60 non-cancerous mucosa tissues (p<0.0001). (b) ROC curve of CD44V expression value to differentiate cancer from non-cancerous mucosa showed high AUC of 0.95, and the most optimal cut-off value was determined to be 1.54 (sensitivity of 0.93 and specificity 0.90, p<0.0001). (c) CD44V expression was significantly higher in right colon cancer than in left colon cancer (p = 0.035). (d) There was no significant difference of CD44V expression according to T factors. (e) There was significant difference of CD44V expression according to N factors (p = 0.04), and CD44V expression was rather reduced in N2 factor. (f) There was no significant difference of CD44V expression according to M factors. (g) Using the cut-off value of 5.71, right colon cancer included 12 cases with high CD44V expression, while left colon cancer had 2 cases with high CD44V expression. In right colon cancer, MSI-H cases were shown in red bars.

We then investigated *CD44V* expression from both primary tumors and corresponding non-cancerous mucosa tissues of the 60 colon cancer patients. *CD44V* expression was significantly increased in primary colon cancer tissues as compared to the corresponding non-cancerous mucosa tissues (p<0.0001) (Fig 2A).

Using ROC (receiver operating characteristics) curve to discriminate tumor tissues from mucosa tissues, area under curve (AUC) was proved to be 0.95 (Fig 2B), and the most optimal cut-off value of the *CD44V* expression level determined in real time RT-PCR was 1.54. Using this optimal cut-off value, *CD44V* expression was high in 92% (55/60) in primary colon cancer tissues, while it was high in 10% (6/60) non-cancerous mucosa tissues (p<0.0001). These data indicated that *CD44V* expression is elevated specifically in primary colon cancer tissues.

We also investigated *CD44T* expression from both primary tumors and corresponding non-cancerous mucosa tissues of the 60 colon cancer patients. *CD44T* expression was significantly increased in primary colon cancer tissues as compared to the corresponding non-cancerous mucosa tissues (p<0.0001) (S1A Fig).

In ROC curve to discriminate tumor tissues from mucosa tissues, AUC was proved to be 0.84 in *CD44T* (S1B Fig), which is inferior to *CD44V* expression, and its optimal cut-off value determined in real time RT-PCR was 5.71. Using this optimal cut-off value, *CD44T* expression was high in 78% (47/60) in primary colon cancer tissues, while it was high in 18% (11/60) non-cancerous mucosa tissues (p<0.0001).

These data indicated that *CD44T* expression is also elevated specifically in primary colon cancer tissues, however its sensitivity and specificity to cancer tissues is much inferior to *CD44V*. This low specificity may be because *CD44T* transcripts included *CD44S* transcripts that were supposed to be expressed in non-cancerous mucosa tissues [21]. Interestingly, nevertheless, *CD44T* expression is closely associated with *CD44V* expression in primary colon cancer (R = 0.62, p<0.0001) as confirmed in CRC cell lines.

### Clinicopathological significance of *CD44V* expression in primary colon cancer

We then examined clinicopathological characteristics of *CD44V* expression by real time RT-PCR in 60 colon cancers. In student’s *t* test, *CD44V* expression was significantly different between right colon and left colon cancer (p = 0.035) (Fig 2C), while there was no statistical difference with regard to other clinicopathological factors such as TNM factors except lymph node metastasis (p = 0.04) (Fig 2D–2F). Unexpectedly, *CD44V* expression rather tended to be low in aggressive colon cancer with lymph node metastasis or distant metastasis.

We then analyzed clinicopathological characteristics after separating right colon cancer from left colon cancer. The most optimal cut-off value of *CD44V* was newly determined to discriminate right colon cancer from left colon cancer by ROC curve analysis. It clarified that the most optimal one was proved to be 6.72, and using this cut-off value, *CD44V* expression was high in 39% (12/31) in right colon cancer, while it was high in 7% (2/29) in left colon cancer (p = 0.004) (Fig 2G).

As high expression of *CD44V* was uniquely seen in right colon cancer, clinicopathological characteristics were again analyzed, limited to right colon cancer (Table 1). As a result, high *CD44V* expression was significantly correlated with young age (p = 0.041) and less lymph node metastasis (p = 0.060).

**Table 1:** Clinicopathological factors and *CD44V* expression in primary right colon cancer.

Variable	Total cases	*CD44V* expression		p-Value
	n = 31	High	Low	
Gender				NS*
male	11 (35%)	4 (33%)	7 (37%)	
female	20 (65%)	8 (67%)	12 (63%)	
Age				p = 0.041*
< 70	9 (29%)	6 (50%)	3 (16%)	
≥ 70	22 (71%)	6 (50%)	16 (84%)	
T factor				NS*
Ⅰ	8 (26%)	5 (41%)	3 (16%)	
Ⅱ	5 (16%)	2 (17%)	3 (16%)	
Ⅲ	15 (48%)	3 (25%)	12 (63%)	
Ⅳ	3 (10%)	2 (17%)	1 (5.0%)	
N factor				NS (p = 0.060)**
0	18 (58%)	10 (83%)	8 (42%)	
1	11 (35%)	2 (17%)	10 (53%)	
2	2 (7.0%)	0 (0%)	1 (5.0%)	
M factor				NS**
0	28 (90%)	12 (100%)	16 (84%)	
1	3 (10%)	0 (0%)	3 (16%)	
Lymphatic invasion				NS*
No	23 (74%)	10 (83%)	13 (68%)	
Yes	8 (26%)	2 (17%)	6 (32%)	
Vascular invasion				NS*
No	11 (35%)	5 (42%)	6 (32%)	
Yes	20 (65%)	7 (58%)	13 (68%)	
MSI				NS (p = 0.077)*
High	8 (26%)	1 (8.0%)	7 (37%)	
Low	23 (74%)	11 (92%)	12 (63%)	

NS: Not Significant

* Chi-square test

** Fisher’s exact test

### *CD44V* expression and microsatellite instability (MSI) in right colon cancer

The unique clinicopathological features of high *CD44V* expression (right colon cancer and younger patients) are reminiscent of phenotypes of microsatellite instability-high (MSI-H) colon cancer. So, we analyzed MSI status using the 5 Bethesda panel markers, and explored the association of high *CD44V* expression with MSI status in right colon cancer. First, MSI-H cases were seen in 8 (26%) out of the 31 right colon cancer, which is consistent with the previous reports (20%~) [22, 23]. In right colon cancer, MSI-H status did not correlate with any clinicopathological factors.

Unexpectedly, MSI-H was rather rarely (1/12, 8%) found in right colon cancer with high *CD44V* expression, as compared with those with low *CD44V* expression (7/19, 37%) (p = 0.077, Table 1, and see red bars of left panel in Fig 2G). This finding is, however, consistent with less aggressive biological features of MSI-H phenotypes.

### Clinicopathological significance of *CD44T* expression in primary colon cancer

We will then describe clinicopathological characteristics of *CD44T* expression by real time RT-PCR in 60 colon cancers. In student’s *t* test, *CD44T* expression was again significantly different between right colon and left colon cancer (p = 0.012) (S1C Fig), while there was no statistical difference with regard to other clinicopathological factors such as TNM factors except distant metastasis (M factor) (p = 0.039) (S1D–S1F Fig). *CD44T* expression tended to be less aggressive colon cancer like *CD44V*, either.

It clarified that the most optimal value to discriminate right colon cancer from left colon cancer was proved to be 7.24, and using this cut-off value, *CD44T* expression was high in 71% (22/31) in right colon cancer, while it was high in 34% (10/29) in left colon cancer (p = 0.005) (S1G Fig). *CD44T* expression did not correlate with MSI-H status in right colon cancer.

### Clinicopathological significance of *CD133* expression in primary colon cancer

**Fig 3:**
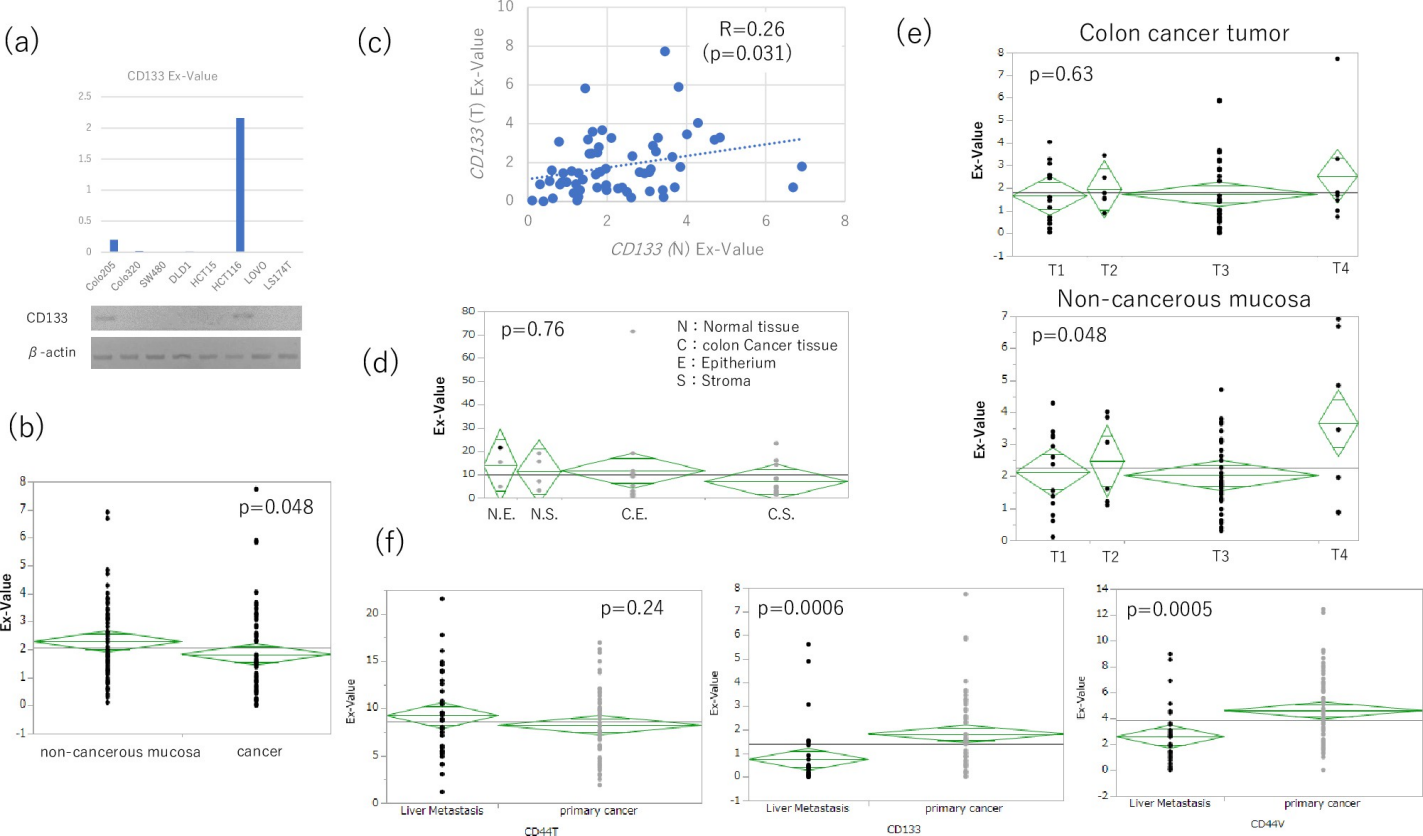
Clinical significance of CD133 expression in primary colon cancer tissues. (a) In semi-quantitative RT-PCR, CD133 transcripts were seen in Colo205 and HCT116 among 8 CRC cell lines (lower panel). Quantitative PCR is consistent with the semi-quantitative RT-PCR (upper panel). (b) CD133 expression was significantly reduced in 60 colon cancer tissues as compared to in 60 non-cancerous mucosa tissues (p = 0.048). (c) CD133 expressions in colon cancer tissues were significantly associated with those in non-cancerous mucosa tissues (R = 0.26, p = 0.026). (d) Public database of microdissection followed by microarray revealed that CD133 expression is rather higher in non-cancerous tissues than in cancer tissues. N.E., normal epithelia N.S., normal stroma C.E., cancer epithelia C.S., cancer stroma (e) CD133 expression in colon cancer tumor tissues was not different according to T factors (upper panel), whereas CD133 expression in non-cancerous mucosa tissues was significantly higher in T4 than in non-T4 (p = 0.048). (f) CD44T expression was not significantly different between primary colon cancer and liver metastasis (p = 0.24, left panel). On the other hand, CD44V expression was significantly lower in liver metastasis than in primary colon cancers (p = 0.0006, middle panel). CD133 expression was significantly lower in liver metastasis than in primary colon cancers, either (p = 0.0005, right panel).

We then investigated *CD133* expression in CRC cell lines, and in both primary tumors and corresponding non-cancerous mucosa tissues of the 60 colon cancer patients. Surprisingly, *CD133* expression was silenced in many CRC cell lines which are tumorigenic like DLD1 cells [24] (Fig 3A), and rather decreased in primary colon cancer tissues as compared to the corresponding non-cancerous mucosa tissues as a whole (p = 0.048) (Fig 3B). Reduced expression of *CD133* in the specific human cancers is, however, consistent with the early papers describing cancer-specific promoter DNA methylation of *CD133* especially in early colorectal cancer [25, 26].

Interestingly, *CD133* expression in colon cancer tissues is weakly correlated with that in the corresponding non-cancerous mucosa tissues (Fig 3C, R = 0.28, p = 0.030), suggesting that *CD133* expression is partially representative of the corresponding non-cancerous mucosa. Microdissection analysis of epithelium and stroma components of both CRC tissues and the corresponding non-cancerous mucosa tissues again recapitulates considerable expression of *CD133* of the non-cancerous mucosa using the public database [27] (Fig 3D), which is consistent with our data.

We then examined clinicopathological characteristics of *CD133* expression by real time RT-PCR in 60 colon cancers. In student’s *t* test, *CD133* expression in the colon cancer tissues was significantly different only in non-cancerous mucosa according to pT factor (p = 0.048) by ANOVA (Fig 3E), while there was no statistical difference with regard to other clinicopathological factors in both tumors and non-cancerous mucosa (S2 Fig).

If the most optimal cut-off value of *CD133* expression to discriminate colon cancer tissues from the corresponding non-cancerous mucosa is selected as 1.20 (AUC = 0.62, p = 0.10), high expression of *CD133* is again significantly associated with right colon cancer, but not with other clinicopathological factors in primary colon tumors (S2 Table).

Finally, we compared expressions of both *CD133* and *CD44V/T* in both liver metastasis of CRC (n = 33) and primary colon tumors (n = 60), and demonstrated that expressions of both *CD133* and *CD44V*, but not *CD44T*, were significantly reduced in liver metastasis as compared to primary colon tumors (Fig 3F). *CD44T* expression was uniquely higher in liver metastasis than in primary colon tumors, however there was no statistical difference.

## Discussion

Cancer stem cell hypothesis has been long focused on because cancer stemness was demonstrated to be functionally associated with drug (treatment) resistance [28–30]. Several subsequent reports proposed that cancer stemness may be correlated even with metastatic ability representing patient poor prognosis [31–33], hence proposing that cancer stemness is considered to be targeted to control cancer metastasis. However, there have remained elusive whether stem cell markers are really predictive of metastatic states of clinical cancers. In this study, we would like to explore this important clinical question. In this study, we did not include NANOG, SOX2, and OCT4, poorly differentiated markers in embryonic stem cells, because poorly differentiated tumors are rare in CRC.

Together with our early reports regarding *Ascl2*, one of the stem cell markers of colon cancer [34, 35], we herein investigated other well-known stem cell markers such as *CD44V* and *CD133* at mRNA level in primary colon cancer. As a result, stem cell markers of *CD44V* showed remarkable increase of their expression in primary colon tumor tissues like *Ascl2* [34, 35], but not associate with aggressive phenotypes of colon cancer at all. These findings suggested that cancer stemness caused by *Ascl2* and *CD44V* does not necessarily represent metastatic potential in clinical samples of the primary tumors.

On the other hand, in this study *CD133* expression is expressed in primary colon tumor tissues, whereas the corresponding non-cancerous mucosa expressed considerable amounts of *CD133* transcripts, either. We wondered if *CD133* mRNA expression is more abundantly expressed in non-cancerous mucosa, because an early report clearly described significant increase of *CD133* expression in colon tumor tissues (p = 0.001) [36]. To validate our confusing finding, we additionally investigated *CD133* mRNA expression in microdissection analysis of CRC using the public database [27], and still confirmed its considerable expression of non-cancerous mucosa epithelium (Fig 3D). Immunohistochemistry by other researchers also supported our finding, because differentiated colon mucosa showed definite expression of CD133 [37]. These findings indicated that a functional role of CD133 may be different between cancer cells and non-cancerous mucosa.

*CD133* mRNA does not correlate with colon cancer metastasis at all in our current study, either. However, our data can not deny the early findings that CD133 protein can correlate with poor survival of CRC [38, 39], because such data was confirmed at protein level differently from our current study. Moreover, CD133 is a well-established cancer stem cell marker and has been definitely demonstrated to play a critical role in treatment resistance against many cancers including colon cancer [28, 40].

HDAC6 (histone deacetylase complex 6), and β-catenin form a ternary complex with CD133 to contextually stabilize β-catenin and CD133 proteins through HDAC6 deacetylase activity to suppress mesenchymal to epithelial differentiation [41]. Thus, CD133 may function as APC/ β-catenin signaling amplifier only in case of transformed colon cells.

*CD133* expression was recently reported to be upregulated in CRC tumors that have a hyperactivated Ras-Raf-MEK-ERK pathway and is therefore related to mutations in *K-ras* or *B-raf* [42]. Moreover, recently *K-ras* mutation was demonstrated to activate cancer stem cells, contributing to colorectal tumorigenesis and metastasis in CRC cells harboring *APC* mutations [43]. These findings can explain why CD133 can play a pivotal role in CRC progression, but not in non-cancerous mucosa.

In our study, nevertheless transcripts expression of stem cell markers did not represent aggressive metastasis at all, as stem cell markers were rather significantly lower in liver metastasis than in primary colon tumors (Fig 3F). Intriguingly, less transcripts of stem cell markers may represent more stromal cells escorts of non-stem tumor cells in metastasis. This mechanism may reach high cost performance during cancer progression, because stem cell population generally occupy a small portion of total tumor cell population [40].

Colon cancer metastasis representing poor prognosis may be associated with specific cancer-associated fibroblast (CAF) harboring unique molecular profiles [44], and stem cell functionality is microenvironmentally defined during tumor expansion [45]. We therefore interpreted our result that transcripts of stem cell markers can be remarkably diluted due to rigorous stromal activation and looked low in metastasis.

CD44V (v8-v10) encompassed v9 which included binding region with cystine transporter xCT to regulate redox status through membrane xCT and promote tumor growth [11], supporting importance of cysteine metabolism in cancer initiation capacity representing cancer stemness [12]. On the other hand, recently soluble CD44 shed from the cancer cell surface promotes tumor metastasis by triggering macrophage IL-1 production in tumor associated macrophage (TAM) [46]. The former can represent CD44V, while the latter may reflect CD44T in this study, postulating a differential role of the individual CD44 isoform status during tumor progression. And *CD44V* expression representing cancer initiation capacity is significantly reduced in aggressive tumor tissues.

Asieh Sadeghi et al. reported that CD44 expression was significantly inversely associated with pathological tumors, lymph nodes, substages of metastasis (pTNM) and lymphatic infiltration [47]. This is in line with our results. Moreover, our finding regarding lymph node involvement is confirmed in a more specified situation (only in right colon cancer). This difference may be due to the evaluation method. Compared to immunohistochemistry, quantitative PCR is considered to be a more objective method for evaluating gene expressions. In this regard, our study is more accurate and objective than that of Asieh Sadeghi’s results. However, our number of patients was much smaller than in their study and we would like to increase our number of patients in the near future. In addition, PCR evaluations of bulky tumors did not consider heterogeneous expression of proteins compared to immunohistochemistry. Nevertheless, similar results obtained from different methodologies were considered to make them reasonable.

We also examined 218 CRC cases in The Cancer Genome Atlas (TCGA) database. In our case, rectal cancer cases were excluded because of their molecular modification by preoperative chemoradiotherapy, whereas rectal cancer was included in left colon cancer in the TCGA database. Although the variant of CD44 is unknown in the TCGA database, CD44 expression was significantly higher in right colon cancer than in left colon cancer as in our current study (S3A Fig). In addition, M factor and CD44 expression tended to be inversely correlated (S3B Fig). These findings support our results.

Recent reports have shown that the interaction of tumor cells with stromal cells such as cancer-related fibroblasts (CAF), tumor-related macrophages, and various forms of T cells, in other words tumor microenvironment (TME), is involved potential tumor progression in distant metastasis [48, 49]. Thus, together with our current study, we interpreted that less demand on stem cell marker-positive cancer cells may characterize metastasis of colon cancer, and believe that TME targeting may be important in controlling tumor metastasis.

In conclusion, our quantitative assessment of cancer stem cell markers revealed their unique expression patterns in right colon cancer, and moreover, we herein concluded that all the stem cell markers (CD44V, CD133, and Ascl2) investigated in our current study can not represent metastatic markers by themselves. This may represent less demand of stemness in terms of cancer metastasis, and TME control might be a bona-fide therapeutic target.

## Supporting information

S1 Table The primer sequences used in the real-time PCR experiments. (DOCX)

S2 Table Clinicopathological factors and *CD133* expression in primary colon cancer. (DOCX)

S1 Fig Clinical significance of CD44T expression in primary colon cancer tissues. (a) CD44T expression is elevated in 60 primary colon cancer tissues as compared to in 60 non-cancerous mucosa tissues (p<0.0001). (b) ROC curve of CD44T expression value to differentiate cancer from non-cancerous mucosa showed high AUC of 0.84, and the most optimal cut-off value was determined to be 5.71 (sensitivity of 0.78 and specificity 0.82, p<0.0001). (c) CD44T expression was significantly higher in right colon cancer than in left colon cancer (p = 0.012). (d) There was no significant difference of CD44T expression according to T factors. (e) There was marginally significant difference of CD44T expression according to N factors (p = 0.10), and CD44T expression was rather reduced in N2 factor. (f) There was significant difference of CD44V expression according to M factors (p = 0.039). CD44T was significantly reduced in M1 than in M0. (g) Using the cut-off value of 7.24, right colon cancer included 22 cases with high CD44V expression, while left colon cancer had 10 cases with high CD44V expression. In right colon cancer, MSI-H cases were shown in red bars.
(PDF)

S2 Fig Clinical significance of CD133 expression in primary colon cancer tissues. (a) There was no significant difference in the expression of *CD133* between right and left primary colon cancer in non cancerous mucosa tissues (p = 0.34). And similar results were obtained for N factors and M factors (p = 0.58 and p = 0.12, respectively). (b) As well as (a), there was no significant difference in the expression of *CD133* between right and left primary colon cancer in colon cancer tumor tissues (p = 0.20) as well as N factors and M factors(p = 0.12 and p = 0.28, respectively).
(PDF)

S3 Fig Clinical significance of CD44 expression in primary colon cancer tissues in the TCGA database. (a) There was significant difference in the expression of CD44 between right and left primary colon cancer (p = 0.00030). (b) M factor and CD44 expression tended to be inversely correlated (p = 0.089).
(PDF)

S1 Raw images (PDF)

S2 Raw images (PDF)
