# Supplementary material for: Less demand on stem cell marker-positive cancer cells may characterize metastasis of colon cancer
Source: PLoS One. 2023 Apr 25;18(4):e0277395. doi: 10.1371/journal.pone.0277395 (PMC10128954; doi:10.1371/journal.pone.0277395)
Supplement: S1 Table — (DOCX) [file pone.0277395.s001.docx]

| Table S1. The primer sequences used in the real-time PCR experiments | |  |
| --- | --- | --- |
| Gene names | Forward primer |  |
|  |  |  |
| *CD44V* (Variant 8-10) | 5'-TGGACAGGACAGGACCTCTT-3' |  |
| *CD44T* (Exon4-5) | 5'-AGTCACAGACCTGCCCAATG-3' |  |
| *CD133 (PROM1)* | 5'-CCTTCATCCACAGATGCTCCTAA-3' |  |
|  | Reverse primer |  |
|  |  |  |
| *CD44V* (Variant 8-10) | 5'-GGGTCTCTTCTTCCACCTGTG-3' |  |
| *CD44T* (Exon4-5) | 5'-AACCTCCTGAAGTGCTGCTC-3' |  |
| *CD133 (PROM1)* | 5'-TAATAAACAGCAGCCCCAGGAC-3' |  |
|  | Probe |  |
|  |  |  |
| *CD44V* (Variant 8-10) | 5'(FAM)-ACGCAGCAGAGTAATTCTCAGAGCTTC-3'(TMRA) |  |
| *CD44T* (Exon4-5) | 5'(FAM)-CCATAACTATTGTTAACCGTGATGGCACCC-3'(TMRA) |  |
| *CD133 (PROM1)* | 5'(FAM)-TGTGGTACAGCCGCGTGATTTCCCAGA-3'(TMRA) |  |
|  | |  |
