# Supplementary material for: Less demand on stem cell marker-positive cancer cells may characterize metastasis of colon cancer
Source: PLoS One. 2023 Apr 25;18(4):e0277395. doi: 10.1371/journal.pone.0277395 (PMC10128954; doi:10.1371/journal.pone.0277395)
Supplement: S2 Table — (DOCX) [file pone.0277395.s002.docx]

| Table S2. Clinicopathological factors and *CD133* expression in primary colon cancer | | | | |
| --- | --- | --- | --- | --- |
| Variable | Total cases | *CD133* expression | | p-Value |
|  | n=60 | High (n=47) | Low (n=13) |  |
| Gender |  |  |  | NS^*^ |
| male | 22 (37%) | 19 (40%) | 3 (23%) |  |
| female | 38 (63%) | 28 (60%) | 10 (77%) |  |
| Age |  |  |  | NS^*^ |
| < 70 | 22 (37%) | 15 (32%) | 7 (54%) |  |
| > 70 | 38 (63%) | 32 (68%) | 6 (46%) |  |
| Location |  |  |  | p=0.020^*^ |
| Right | 31 (52%) | 28 (60%) | 3 (23%) |  |
| Left | 29 (48%) | 19 (40%) | 10 (77%) |  |
| T factor |  |  |  | NS^*^ |
| 1 | 13 (22%) | 9 (18%) | 4 (31%) |  |
| 2 | 6 (10%) | 5 (11%) | 1 (8.0%) |  |
| 3 | 34 (57%) | 28 (60%) | 6 (46%) |  |
| 4 | 7 (11%) | 5 (11%) | 2 (15%) |  |
| N factor |  |  |  | NS^*^ |
| 0 | 38 (63%) | 30 (64%) | 8 (61%) |  |
| 1 | 18 (30%) | 14 (30%) | 4 (31%) |  |
| 2 | 4 (7.0%) | 3 (6.0%) | 1 (8.0%) |  |
| M factor |  |  |  | NS^**^ |
| 0 | 54 (90%) | 41 (87%) | 13 (100%) |  |
| 1 | 6 (10%) | 6 (13%) | 0 (0.0%) |  |
| Lymphatic invasion |  |  |  | NS^*^ |
| No | 44 (73%) | 36 (77%) | 8 (61%) |  |
| Yes | 16 (27%) | 11 (23%) | 5 (39%) |  |
| Vascular invasion |  |  |  | NS^*^ |
| No | 19 (32%) | 17 (36%) | 2 (15%) |  |
| Yes | 41 (68%) | 30 (64%) | 11 (85%) |  |
| NS : Not Significant 　* Chi-square test　** Fisher's exact test | | | | |
