# Supplementary material for: Less demand on stem cell marker-positive cancer cells may characterize metastasis of colon cancer
Source: PLoS One. 2023 Apr 25;18(4):e0277395. doi: 10.1371/journal.pone.0277395 (PMC10128954; doi:10.1371/journal.pone.0277395)
Supplement: S1 Fig — (a) CD44T expression is elevated in 60 primary colon cancer tissues as compared to in 60 non-cancerous mucosa tissues (p<0.0001). (b) ROC curve of CD44T expression value to differentiate cancer from non-cancerous mucosa showed high AUC of 0.84, and the most optimal cut-off value was determined to be 5.71 (sensitivity of 0.78 and specificity 0.82, p<0.0001). (c) CD44T expression was significantly higher in right colon cancer than in left colon cancer (p = 0.012). (d) There was no significant difference of CD44T expression according to T factors. (e) There was marginally significant difference of CD44T expression according to N factors (p = 0.10), and CD44T expression was rather reduced in N2 factor. (f) There was significant difference of CD44V expression according to M factors (p = 0.039). CD44T was significantly reduced in M1 than in M0. (g) Using the cut-off value of 7.24, right colon cancer included 22 cases with high CD44V expression, while left colon cancer had 10 cases with high CD44V expression. In right colon cancer, MSI-H cases were shown in red bars. (PDF) [file pone.0277395.s003.pdf]

Fig. S1

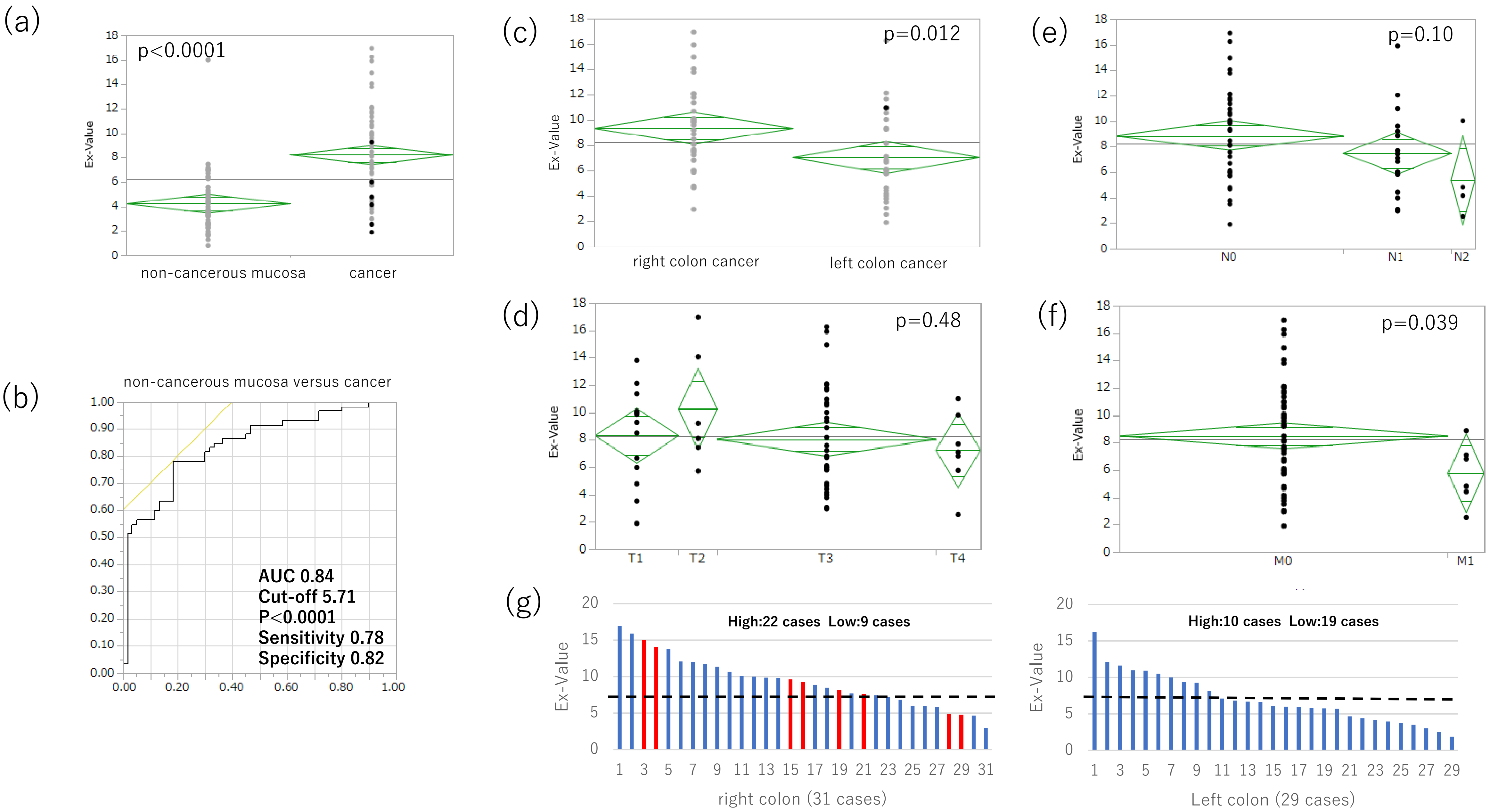

(a)
p<0.0001
Ex-Value
non-cancerous mucosa
cancer
(b)
non-cancerous mucosa versus cancer
AUC 0.84
Cut-off 5.71
P<0.0001
Sensitivity 0.78
Specificity 0.82
(c)
p=0.012
Ex-Value
right colon cancer
left colon cancer
(d)
p=0.48
Ex-Value
T1
T2
T3
T4
(e)
p=0.10
Ex-Value
N0
N1
N2
(f)
p=0.039
Ex-Value
M0
M1
(g)
High:22 cases Low:9 cases
Ex-Value
right colon (31 cases)
High:10 cases Low:19 cases
Ex-Value
Left colon (29 cases)

Fig. S1 Clinical significance of CD44T expression in primary colon cancer tissues.

(a) CD44T expression is elevated in 60 primary colon cancer tissues as compared to in 60 non-cancerous mucosa tissues ($p<0.0001$).

(b) ROC curve of CD44T expression value to differentiate cancer from non-cancerous mucosa showed high AUC of 0.84, and the most optimal cut-off value was determined to be 5.71 (sensitivity of 0.78 and specificity 0.82, $p<0.0001$).

(c) CD44T expression was significantly higher in right colon cancer than in left colon cancer ($p=0.012$).

(d) There was no significant difference of CD44T expression according to T factors.

(e) There was marginally significant difference of CD44T expression according to N factors ($p=0.10$), and CD44T expression was rather reduced in N2 factor.

(f) There was significant difference of CD44V expression according to M factors ($p=0.039$). CD44T was significantly reduced in M1 than in M0

(g) Using the cut-off value of 7.24, right colon cancer included 22 cases with high CD44V expression, while left colon cancer had 10 cases with high CD44V expression. In right colon cancer, MSI-H cases were shown in red bars.
