# Supplementary material for: Less demand on stem cell marker-positive cancer cells may characterize metastasis of colon cancer
Source: PLoS One. 2023 Apr 25;18(4):e0277395. doi: 10.1371/journal.pone.0277395 (PMC10128954; doi:10.1371/journal.pone.0277395)
Supplement: S2 Fig — (a) There was no significant difference in the expression of CD133 between right and left primary colon cancer in non cancerous mucosa tissues (p = 0.34). And similar results were obtained for N factors and M factors (p = 0.58 and p = 0.12, respectively). (b) As well as (a), there was no significant difference in the expression of CD133 between right and left primary colon cancer in colon cancer tumor tissues (p = 0.20) as well as N factors and M factors(p = 0.12 and p = 0.28, respectively). (PDF) [file pone.0277395.s004.pdf]

Fig. S2

(a) Non cancerous mucosa

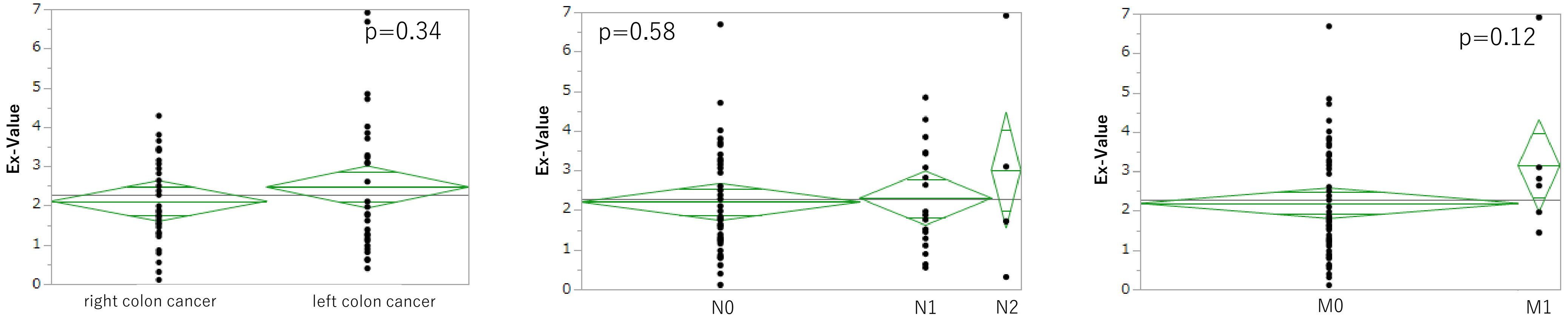

(b) Colon cancer tumor

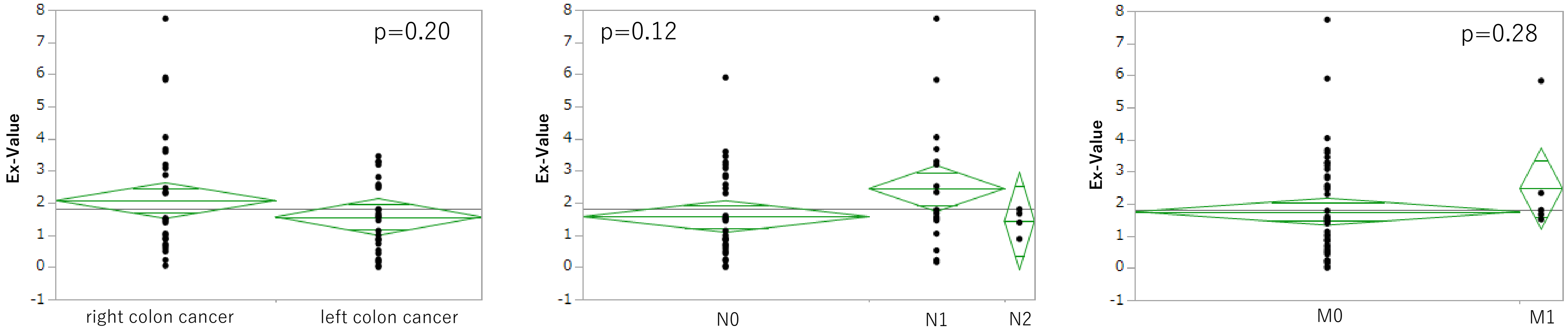

Fig.S2

(a) There was no significant difference in the expression of *CD133* between right and left primary colon cancer in non cancerous mucosa tissues (p=0.34).

And similar results were obtained for N factors and M factors (p=0.58 and p=0.12, respectively).

(b) As well as (a), there was no significant difference in the expression of *CD133* between right and left primary colon cancer in colon cancer tumor tissues (p=0.20) as well as N factors and M factors(p=0.12 and p=0.28, respectively).
