# Supplementary material for: Less demand on stem cell marker-positive cancer cells may characterize metastasis of colon cancer
Source: PLoS One. 2023 Apr 25;18(4):e0277395. doi: 10.1371/journal.pone.0277395 (PMC10128954; doi:10.1371/journal.pone.0277395)
Supplement: S3 Fig — (a) There was significant difference in the expression of CD44 between right and left primary colon cancer (p = 0.00030). (b) M factor and CD44 expression tended to be inversely correlated (p = 0.089). (PDF) [file pone.0277395.s005.pdf]

Fig. S3

(a)

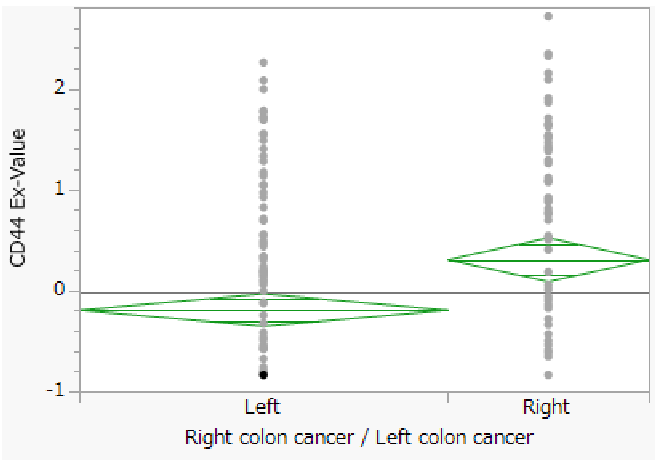

(b)

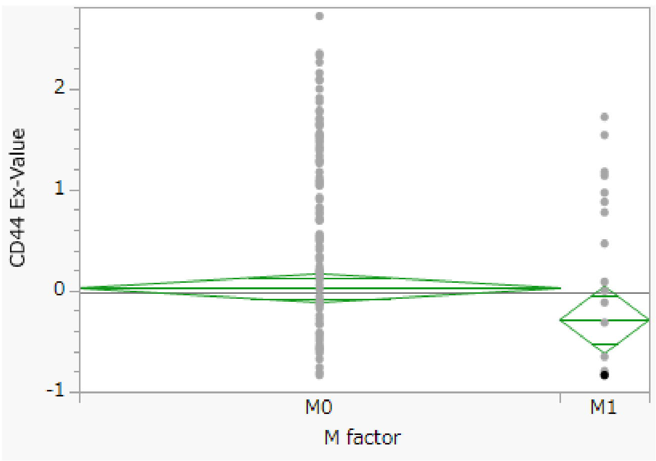

(a) There was significant difference in the expression of CD44 between right and left primary colon cancer (p=0.00030).
(b) M factor and CD44 expression tended to be inversely correlated (p=0.089).
