# Supplementary figures and images for: Less demand on stem cell marker-positive cancer cells may characterize metastasis of colon cancer
Source: PLoS One. 2023 Apr 25;18(4):e0277395. doi: 10.1371/journal.pone.0277395 (PMC10128954; doi:10.1371/journal.pone.0277395)

## Photos of Gel in RT-PCR

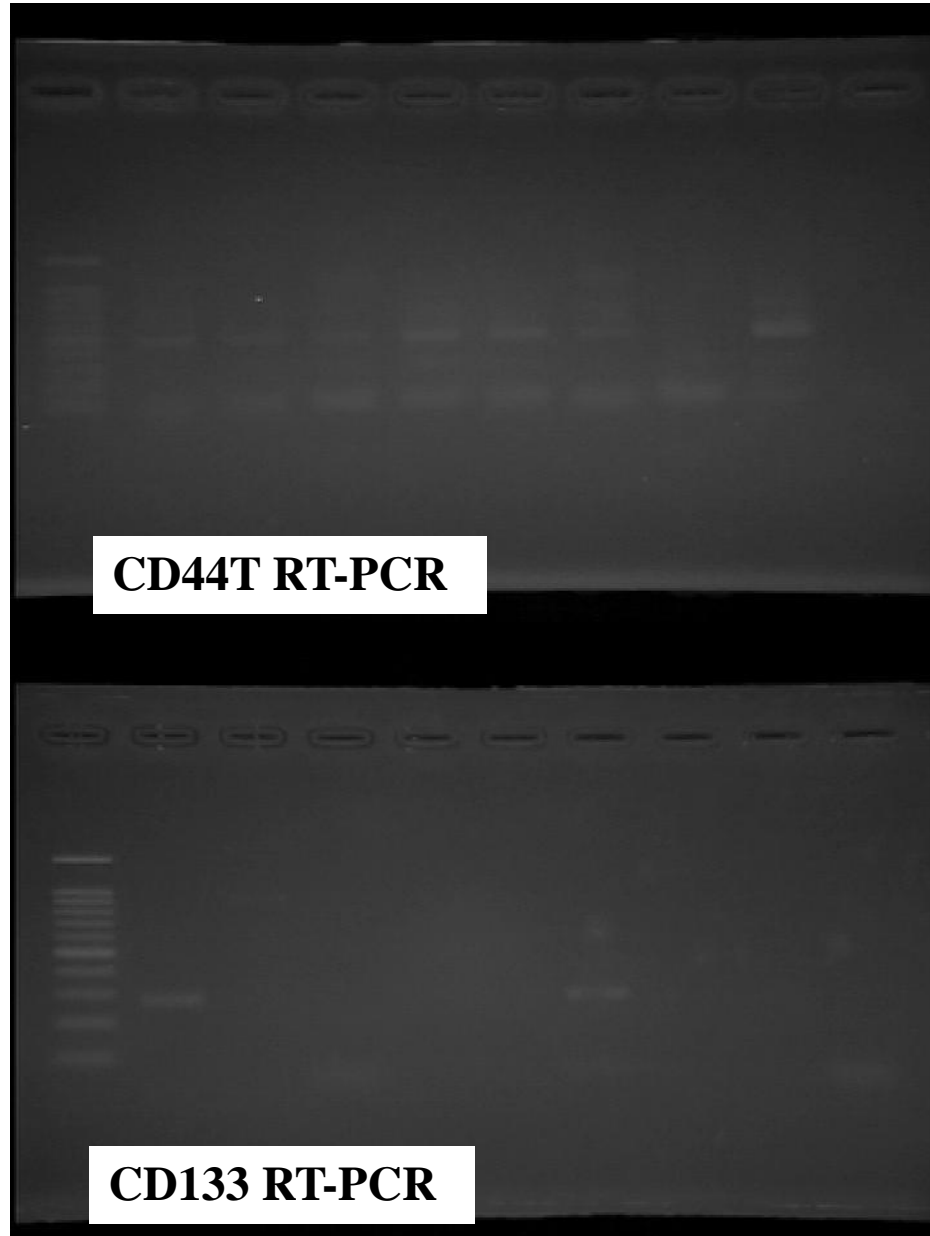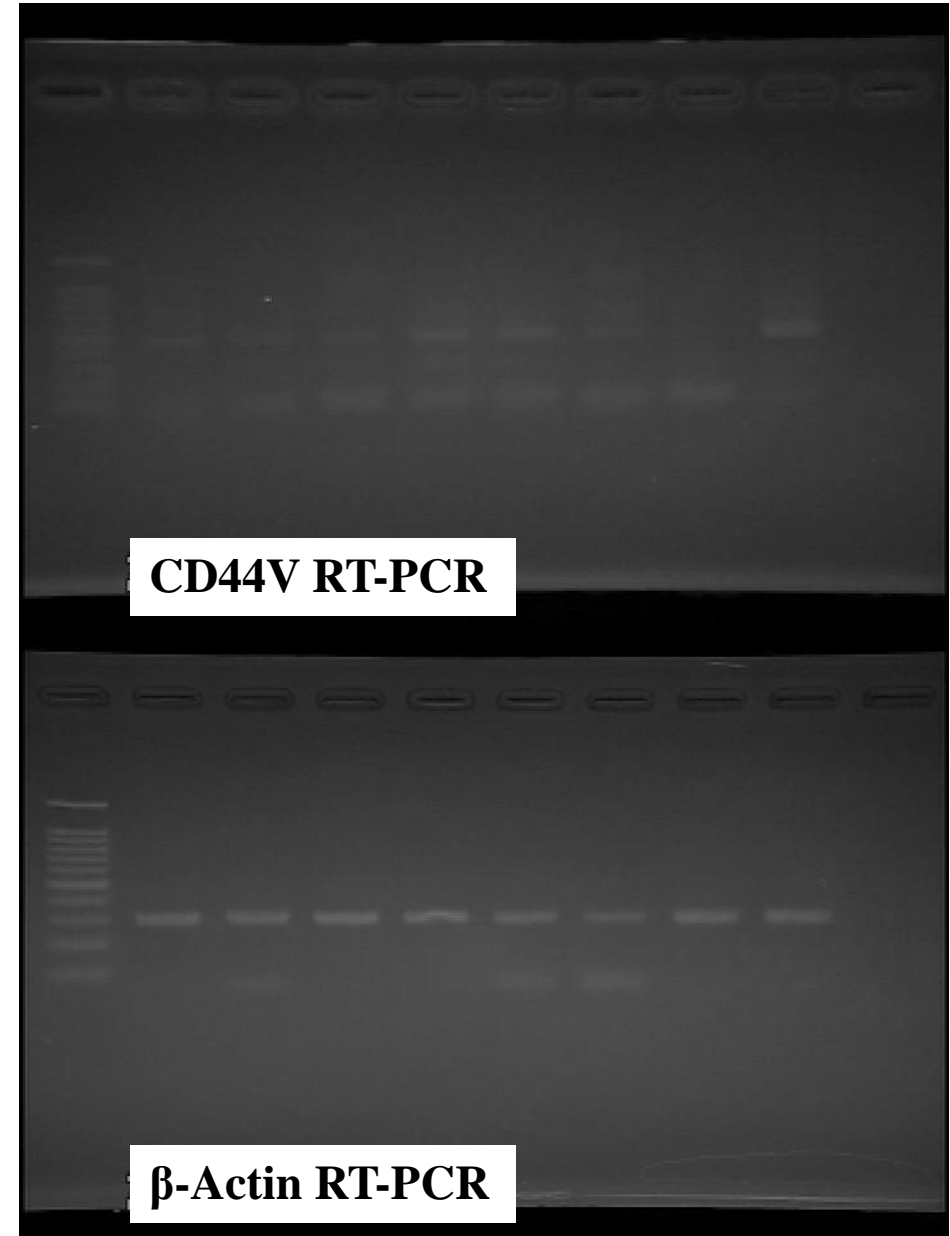

Supplement: S1 Raw images — (PDF) [file pone.0277395.s006.pdf]
