# Supplementary material for: Less demand on stem cell marker-positive cancer cells may characterize metastasis of colon cancer
Source: PLoS One. 2023 Apr 25;18(4):e0277395. doi: 10.1371/journal.pone.0277395 (PMC10128954; doi:10.1371/journal.pone.0277395)
Supplement: S2 Raw images — (PDF) [file pone.0277395.s007.pdf]

Case	CD44V Ex-Value (Normal Tissue)	CD44V Ex-Value (Tumor Tissue)	CD44T Ex-Value (Normal Tissue)	CD44T Ex-Value (Tumor Tisuue)	CD133 Ex-Value (Normal Tissue)	CD133 Ex-Value (Tumor Tissue)	Age	Sex	Tumor Site	Stage(UICC8th)	Dukes	Tissue type	Tumor Size (cm)	Depth	ly	v	INF	Regional lymph node metastasis (number)	Preoperative distant metastasis (o:None)	Preoperative Liver metastasis (o:None)
1	2.76	7.454545455	6.403013183	10.95092025	0.395424837	0.008718182	79	M	S	ⅡA	B	tub2	7.5×5.0	pT3(SS)	0	2	b	0	0	0
3	2.105691057	4.3046875	4.420677362	8.168761221	0.97826087	0.9958159	62	M	S	ⅡA	B	tub1	1.5×1.5	pT3	1	2	b	0	0	0
4	0.187635575	12.44444444	5.295454545	10	2.605504587	0.203623188	50	F	C	0	A	tub1	4.0×2.5	pTis(M)	0	0		0	0	0
5	0.391478029	1.366336634	2.567493113	3.047619048	0.628762542	0.160757315	65	F	S	ⅢB	C	tub2	7.0×5.5	pT3	0	2	b	3	0	0
6	1.398135819	6.576086957	3.767705382	12.13307241	1.555051078	2.457711443	89	F	S	Ⅰ	A	tub1	1.5×1.5	pT1a(SM)	0	0	b	0	0	0
9	1.330935252	7.682539683	6.25	10.99391481	0.892655367	1.462686567	55	F	S	ⅢB	C	tub2	3.3×4.5	pT4a(SE)	2	1	b	3	0	0
10	1.336696091	4.601677149	4.89010989	10.67567568	2.105263158	3.26984127	74	M	T	ⅡA	B	pap	2.5×2.5	T3	0	0		0	0	0
11	1.058333333	6.788432268	7.207446809	8.49122807	3.28313253	3.28009828	75	F	A	0	A	tub1	5.9×3.9	pTis(M)	0	0		0	0	0
12	0.984848485	3.299212598	4.290976059	6.11328125	1.975609756	0.846715328	69	M	S	ⅡA	B	tub1	3.3×3.1	pT3	1	2	b	0	0	0
13	0.309438471	4.538461538	2.303977273	9.209302326	1.105341246	1.566110398	70	F	A	ⅢA	C	tub2	4.5×2.6	pT2(MP)	0	2	a	1	0	0
14	1.198630137	2.315789474	3.691983122	4.662162162	1.243386243	0.044557823	63	F	A	ⅡA	B	tub2	5.5×5.0	pT3(SS)	0	1	b	0	0	0
15	1.04587156	5.35	4.655581948	15.91603053	1.281045752	1.459119497	75	M	A	ⅢB	C	tub2,pap	4.3×4.1	pT3(SS)	0	1	b	2	0	0
16	1.191666667	1.544871795	3.31641286	3.983739837	0.548076923	1.044444444	55	F	S	ⅢB	C	tub2>pap	3.0×2.8	pT3(SS)	0	1	b	1	0	0
17	0.781065089	6.19047619	3.356741573	10.08403361	1.156996587	0.429032258	83	F	T	Ⅰ	A	tub2>muc	1.4×1.3	pT1b(SM)	0	1	b	0	0	0
18	0.643564356	3.168224299	3.658892128	6.018735363	1.874045802	3.676646707	71	F	A	ⅢB	C	tub2	3.2×2.3	pT3(SS)	0	1	b	1	0	0
19	0.970930233	6.716535433	4.32	9.871520343	1.384976526	1.136029412	72	M	A	Ⅰ	A	tub1>pap	4.0×2.7	pT1b(SM)	0	0	b	0	0	0
20	0.452781371	1.619402985	3.214285714	3.777777778	1.317567568	0.58436214	71	M	S	ⅡA	B	tub1	3.0×2.5	pT3(SS)	0	1	b	0	0	0
21	1	9.28030303	3.469387755	11.35542169	0.607142857	1.6	86	F	Appe	Ⅰ	A	tub2	4.5×4.0	pT1b(SM,2500μm)	0	1	b	0	0	0
22	1.294032023	1.991803279	6.734177215	5.714285714	1.215517241	0.899082569	71	M	S	Ⅰ	A	tub2	4.5×3.2	pT2(MP)	0	0	b	0	0	0
24	1.237762238	2.365853659	6.940700809	9.360824742	0.810035842	0.872340426	40	F	S	ⅡA	B	tub2	4.4×4.3	pT3(SS)	0	2	b	0	0	0
25	1.496815287	4.776859504	5.103189493	16.24423963	3.801724138	5.899053628	82	F	S	ⅡA	B	tub2	3.5×3.0	pT3(SS)	0	1	b	0	0	0
27	0.769633508	5.317982456	5.559006211	11.64179104	1.275395034	0.248357424	73	F	S	ⅡA	B	tub1	3.7×3.0	pT3(SS)	0	0	b	0	0	0
28	0.320072333	3.721910112	4.23880597	10.02	0.308730159	0.88028169	42	F	S	ⅢB	C	tub2	3.2×3.2	pT3(SS)	3	2	b	6	0	0
29	1.322580645	1.273333333	7.496038035	7.236024845	1.634782609	3.594095941	77	F	A	ⅡA	B	muc	1.1×1.0	pT3(SS)	0	0	b	0	0	0
30	1.145038168	1.782312925	6.457990115	7.45526839	3.846153846	1.78343949	75	F	A	ⅢA	C	tub2	2.0×2.0	pT2(MP)	0	2	b	1	0	0
31	0.954545455	8.270042194	7.083333333	12.04152249	1.753623188	2.523364486	68	F	T	ⅢB	C	tub1>tub2	3.0×2.5	pT3(SS)	1	0	b	1	0	0
32	2.473282443	2.207792208	7.07201889	5.789473684	1.831932773	1.527559055	86	F	S	ⅡA	B	tub2	2.5×2.1	pT3(SS)	0	1	b	0	0	0
33	0.865079365	3.599160546	6.993464052	8.098765432	1.612244898	2.473053892	60	F	T	Ⅰ	A	tub1	1.8×1.4	pT2(MP)	0	0	b	0	0	0
34	1.413793103	0.019428571	4.664031621	4.815498155	3.1	1.670807453	81	M	T	ⅣA	D	tub2	4.5×4.5	pT3(SS)	1	2	b	5	1	1
35	1.095652174	8.056537102	5.305263158	16.94594595	3.064516129	1.507575758	75	M	T	Ⅰ	A	tub1>tub2	2.4×3.5	pT2(MP)	0	1	b	0	0	0
36	0.93125	7.908496732	5.609243697	13.78752887	3.229461756	2.574002574	62	F	T	0	A	pap>tub1	4.5×3.3	pTis(M)	0	0		0	0	0
37	0.751552795	3.097087379	5.632582322	14.94475138	3.705882353	0.732692308	90	M	A	ⅡA	B	tub1>tub2	5.5×4.0	pT3(SS)	0	1	b	0	0	0
38	0.695104895	7.060344828	3.986636971	12.09359606	3.153439153	2.86809816	67	F	A	ⅡA	B	tub1>pap	5.5×4.5	pT3(SS)	0	1	b	0	0	0
39	0.935960591	2.801724138	4.479495268	8.885869565	2.816993464	1.516736402	80	F	A	ⅣA	D	por1	5.5×4.0	pT3(SS)	2	2	b	1	1	0
40	0.95505618	4.607407407	3.585185185	10.53061224	1.787878788	2.8	53	M	S	ⅡA	B	tub2>tub1>pap	6.0×3.5	pT3(SS)	0	3	b	0	0	0
41	0.531707317	2.299107143	3.532110092	7.113821138	1.966386555	1.681957187	73	F	D	ⅣA	D	tub2>por	2.5×4.0	pT4a(SE)	0	1	b	1	1	1
42	2.890365449	1.616071429	15.99067599	9.603283174	3.076923077	0.524157303	86	F	A	ⅢB	C	muc	10.5×4.5	pT3(SS)	1	0		2	0	0
43	1.162337662	4.022988506	2.267241379	6.672504378	0.786982249	3.082089552	67	F	S	Ⅰ	A	tub2	1.4×1.4	pT1b(SM,1500μm)	1	0	b	0	0	0
44	1.335740072	8.653637351	3.264	11.77536232	4.710526316	3.184615385	63	F	T	ⅡA	B	tub2	3.1×2.7	pT3(SS)	0	1	b	0	0	0
45	1.055888224	4.774590164	4.75	5.961538462	1.747052519	0.720588235	66	M	D	ⅡA	B	muc>pap>tub2	11.0×8.2	pT3(SS)	0	1	b	0	0	0
46	0.665048544	6.376068376	2.453416149	6.71	2.274509804	0.664473684	67	F	S	ⅡA	B	tub1	4.7×3.0	pT3(SS)	0	1	b	0	0	0
47	1.52	5.254716981	4.282608696	6.825806452	1.44516129	5.832167832	45	M	D	ⅣA	D	tub2	2.6×2.3	pT3(SS)	0	2	b	1	1	1
48	1.318452381	6.123348018	3.262158956	5.974477958	4.289473684	4.043956044	74	F	A	ⅢA	C	tub2	4.7×2.6	pT1b(SM)	2	3	b	2	0	0
49	1.540913921	3.069873998	3.554153523	4.666666667	3.645454545	2.298245614	70	F	S	ⅡA	B	tub1	7.5×5.5	T3	0	2	b	0	0	0
50	0.615226337	5.572139303	3.52303523	7.576923077	1.986970684	0.588	82	F	T	ⅡA	B	tub2	8.0×7.0	T3	0	2		0	0	0
51	1.07486631	1.704545455	1.61722488	3.527607362	0.109195402	0.048803419	73	F	S	Ⅰ	A	tub2	3.2×2.8	pT1b(SM)	0	1	b	0	0	0
52	0.755	2.757078987	0.804232804	5.76056338	0.862765957	0.998989899	85	M	S	ⅡB	B	tub1	2.7×1.7	pT4a(SE)	1	0	b	0	0	0
53	1.23	12.18461538	2.254098361	14.05797101	4.012219959	3.454545455	48	M	T	Ⅰ	A	tub1	3.3×2.7	pT2(MP)	0	1	b	0	0	0
54	0.170629371	2.2	1.783333333	5.829228243	1.517110266	3.187772926	78	M	A	ⅢB	C	tub1	4.5×3.0	pT3	1	1	b	1	0	0
55	0.41322314	1.00621118	2.511848341	2.947826087	3.421052632	0.226573427	87	F	A	ⅢB	C	por1	11.5×7.0	pT3	1	0	a	1	0	0
56	0.546753247	3.552915767	2.615384615	6.82328907	4.842883549	3.290780142	73	M	A	ⅣA	D	tub2	4.0×4.0	pT4a(SE)	0	1	b	1	0	0
57	0.794019934	5.088105727	2.906976744	4.42408377	2.63751763	2.334004024	83	F	S	ⅢB	C	tub2	6.2×3.6	pT3	0	1	b	3	1	0
58	1.029411765	6.743718593	2.62541806	9.816971714	6.688311688	0.733449477	70	F	A	ⅡB	B	tub1	6.4×5.0	pT4a(SE)	0	1	b	0	0	0
59	0.71686747	9.111675127	3.672727273	7.695716396	3.457446809	7.731481481	76	M	A	ⅢB	C	tub1	4.5×4.0	pT4a(SE)	1	2	b	2	0	0
60	0.841105354	5.536585366	1.283834586	6.007575758	2.5	0.492662474	71	F	D	ⅡA	B	tub2	3.5×2.2	pT3	1	0	b	0	0	0
61	0.939597315	1.6443769	1.970046083	4.161290323	1.71314741	1.38961039	72	M	S	ⅢC	C	tub2	5.5×3.7	pT3	0	1	b	8	0	0
62	1.270886076	3.18647541	1.70703125	4.788571429	2.942008487	1.450189155	68	M	T	Ⅰ	A	tub2	2.0×1.8	pT1b	0	0	b	0	0	0
63	2.095588235	1.92139738	2.674650699	1.907600596	3.403614458	0.588100686	72	M	S	Ⅰ	A	tub2	1.5×1.0	pT1b	0	0	b	0	0	0
64	0.965909091	6.040816327	1.732954545	9.276895944	2.379213483	0.717352415	53	F	S	Ⅰ	A	tub2	1.4×0.8	pT1b	0	0	b	0	0	0
83	1.582952816	2.10617284	3.252427184	2.529832936	6.912621359	1.804819277	80	F	D	ⅣA	C	tub1	4.0×4.0	pT4a	1	1	b	5	1	1
